# Draft Genome Sequence of Endophytic Sphingomonas faeni Strain ALB2, Isolated from the Leaf of a Cold-Desert Medicinal Plant

**DOI:** 10.1128/mra.00687-22

**Published:** 2022-10-13

**Authors:** Priyanka Bhardwaj, Rahul Jain, Sanjay Kumar

**Affiliations:** a Biotechnology Division, CSIR-Institute of Himalayan Bioresource Technology, Palampur, Himachal Pradesh, India; b Academy of Scientific and Innovative Research, Ghaziabad, Uttar Pradesh, India; University of Maryland School of Medicine

## Abstract

A leaf endophyte, Sphingomonas faeni strain ALB2, was isolated from a high-altitude medicinal plant, Arnebia euchorma. The draft genome sequence of the bacterium comprised 4,720,245 bp and contained 4,233 protein-coding genes, with a GC content of 65.66%. The bacterium harbored numerous biotechnology-relevant genes in its genome.

## ANNOUNCEMENT

Characterized by the presence of glycosphingolipids in their outer membranes, *Sphingomonas* strains are integral components of soil and plant microbiomes (1, 2). Many species of *Sphingomonas* display plant-beneficial activities (3, 4) and confer bioremediation in soil (5). During ongoing work on microbial endophytes, we identified a bacterial strain, ALB2, with closest similarity to Sphingomonas faeni strain MA-olki (GenBank accession number AJ429239) on the basis of 16S rRNA gene sequence analysis using the EzTaxon database. The bacterium was isolated as an endophyte from the leaf tissues of the medicinal plant Arnebia euchorma, growing in the cold-desert soil (altitude, 4,254 m above sea level; coordinates, 32°16′27.93″N, 78°4′27.23″E) in the Lahaul and Spiti district of Himachal Pradesh, India (2). The genome of this bacterium was sequenced to further explore its genetic features and metabolic capabilities.

For genomic library preparation, next-generation sequencing (NGS)-grade genomic DNA was extracted from ALB2 after bacteria were grown in lysogeny broth for 24 h at 20°C, as described by Salvà-Serra et al. (6). The genomic DNA was quantified with the QuantiFluor ONE double-stranded DNA (dsDNA) system (Promega Corp., USA), and 100 ng DNA was used to prepare a genomic library using the QIAseq FX DNA library kit (Qiagen, USA) according to the manufacturer’s instructions. The paired-end NGS library, with an average size of ~450 bp, was sequenced on the Illumina NovaSeq 6000 platform (2 × 150 bp). The sequencing generated 14,169,410 reads. The quality control, *de novo* assembly, and annotations were performed using the Bactopia pipeline v2.1.0 (7). The quality of the raw reads was evaluated using FastQC v0.11.9. The final analysis included 4,949,222 high-quality reads with an average Phred score of 36.

**FIG 1:**
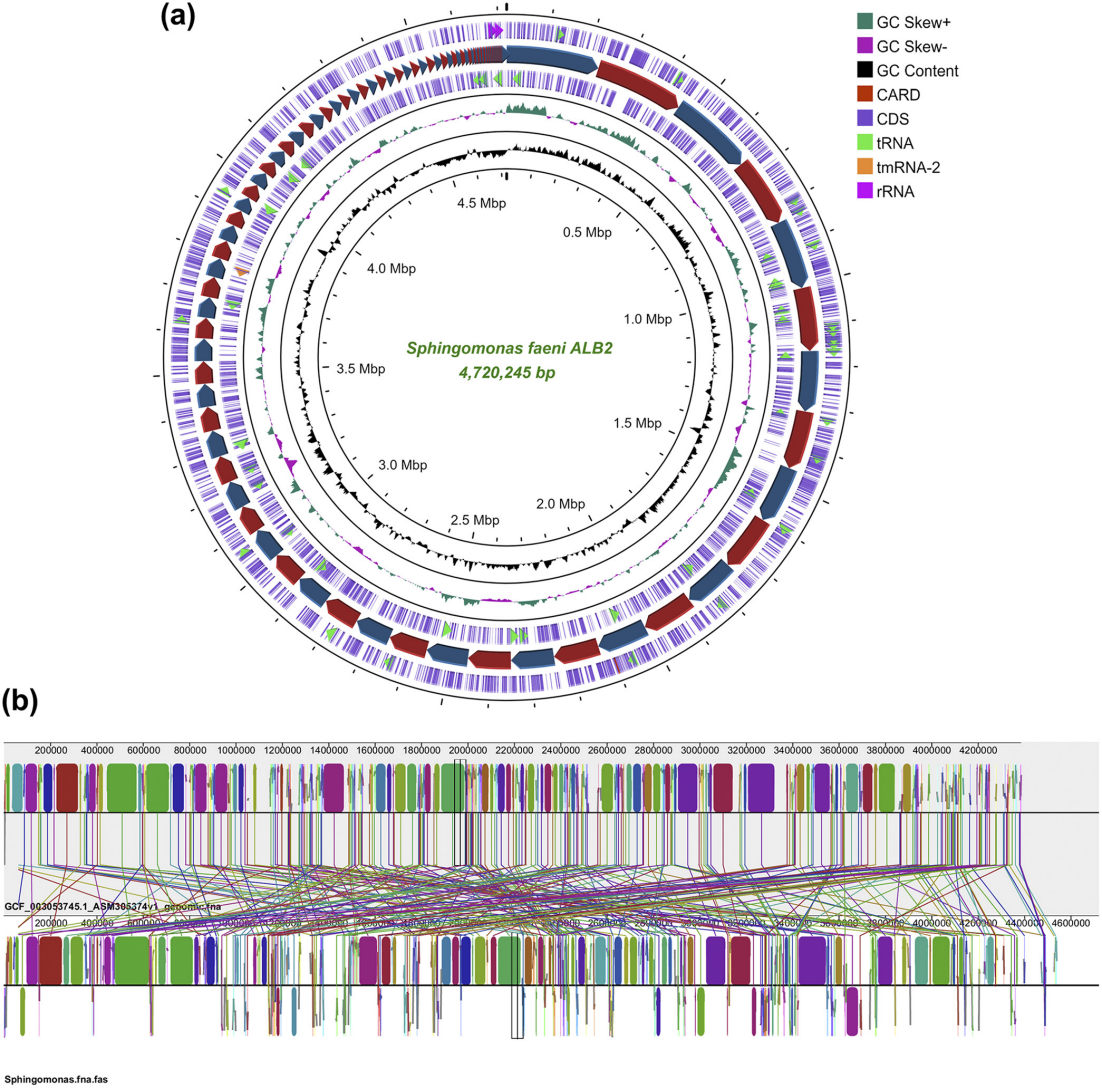
Circular chromosomal map and Mauve alignment of the draft genome of Sphingomonas faeni strain ALB2. (a) Chromosomal map of the draft genome (4,720,245 bp) of ALB2, showing DNA coding sequences (CDSs), tRNAs, rRNAs, tmRNAs, GC skew, GC content, and the Comprehensive Antibiotic Resistance Database (CARD) resistance gene identifier (RGI). (b) Mauve alignment of the ALB2 draft genome with the reference genome of S. faeni strain MA-Olki (GenBank assembly accession number GCA_003053745.1).

QUAST (8) analysis indicated that the draft genome consisted of 105 contigs of ≥500 bp (minimum size, 666 bp; maximum size, 230,795 bp; *N*_50_, 106,428 bp; *L*_50_, 16). The final assembly consisted of 4,720,245 bp, with a coverage depth of 110× and a GC content of 65.66%. Average nucleotide identity (ANI) (FastANI) analysis performed with DFAST (9) showed the closest similarity of ALB2 to Sphingomonas albertensis strain DOAB 1063 (GenBank assembly accession number GCA_014358075.1) (ANI of 91.99%) and S. faeni strain MA-Olki (GenBank assembly accession number GCA_003053745.1) (ANI of 88.78%). The circular chromosome was generated using the Proksee server (Fig. 1a). The contigs in the draft genome were reordered and aligned with the reference genome of S. faeni strain MA-Olki (GenBank accession number QAYE00000000.1) using the Mauve v20150226 software tool (10) (Fig. 1b).

Prokka (11) analysis predicted 4,288 genes, 4,233 coding sequences, 55 RNA genes (of which 50 are tRNAs, 4 are rRNAs, and 1 is a transfer-messenger RNA [tmRNA]). Putative functions were assigned to 2,127 protein-coding genes. Genome analysis revealed that ALB2 contained several genes associated with plant growth promotion, abiotic stress alleviation, temperature adaptation, production of hydrolytic enzymes, and heavy metal bioremediation (Table 1). The genomic information provided for S. faeni strain ALB2 can help to modify bacterial genetics for deciphering the molecular mechanisms of plant-bacterium interactions.

**TABLE 1:** Putative functions of different protein-coding genes identified in the genome of Sphingomonas faeni strain ALB2

Putative functionality and protein(s)	Associated gene(s)
Phosphorus availability	
Pyrroloquinoline quinone biosynthesis	*pqqB*, *pqqC*, *pqqD*, *pqqE*
Alkaline phosphatase D	*phoD*
Polyphosphate kinase	*ppk*
Exopolyphosphatase	*ppx*
Sulfur availability and transport	
Alkanesulfonate monooxygenase	*ssuD*
Methanesulfonate monooxygenase	*msuD*
NADPH-dependent flavin mononucleotide reductase ArsH	*arsH*
Indole acetic acid biosynthesis	
Indole-3-glycerol phosphate synthase	*trpC*
Tryptophan synthase	*trpA*, *trpB*
Salicylic acid biosynthesis	
Isochorismate synthase	*menF*
Chorismate synthase	*aroC*
1-Aminocyclopropane-1-carboxylate deaminase production	
Acetyl-coenzyme A carboxylase carboxyl transferase	*accA*, *accD*
Oxidative stress and abiotic stress tolerance	
Glutathione *S*-transferase	*gst*, *gstB*
Hydroxyacylglutathione hydrolase	*gloB*, *gloC*
Glutathione synthetase	*gshB*
Superoxide dismutase	*sodA*, *chrC*
Polyphenol oxidase	*yfiH*
Catalases	*katE*, *katG*
Trehalose-6-phosphate synthase, phosphatase	*ostA*, *ostB*
Betaine aldehyde dehydrogenase	*gbsA*
Proline/betaine transporter	*proP*
Hydrolytic enzymes	
Phospholipase D	*pld*
α-Amylase	*amy*
Coccaine esterase	*cocE*
Proteases	*mmpA*, *pmbA*, *tldD*, *htpX*, *sppA*
Temperature adaptation	
Cold shock protein	*cspA*
Small heat shock protein	*ibpA*
Heat shock protein	*hspQ*
Heat-inducible transcription repressor	*hrcA*
Heavy metal bioremediation	
Cobalt-zinc-cadmium resistance protein	*czcA*, *czcB*, *czcC*, *czcD*
Nickel and cobalt resistance protein	*cnrA*
Arsenate reductase	*arsB*, *arsC*
Copper resistance protein	*copC*, *copD*

### Data availability.

The draft genome of Sphingomonas faeni strain ALB2 (GenBank accession number JANBVH000000000) and the associated raw sequence reads (SRA accession number SRR19970817) were deposited in GenBank under BioProject accession number PRJNA855357 and BioSample accession number SAMN29490045. The version described in this paper is version JANBVH010000000. The 16S rRNA gene sequence of the bacterium is available under GenBank accession number MW665326.
